# Prognostic significance of G2/M arrest signaling pathway proteins in advanced non-small cell lung cancer patients

**DOI:** 10.3892/ol.2015.2842

**Published:** 2015-01-02

**Authors:** JING WANG, YUHAI ZHANG, SHUDI XU, WEIJIE LI, ZHANGQIN CHEN, ZHE WANG, XINPENG HAN, YILING ZHAO, SHENGQING LI

**Affiliations:** 1 Department of Pulmonary and Critical Care Medicine, Xijing Hospital, Xi’an, Shaanxi, P.R. China; 2 Department of Medical Statistics, Fourth Military Medical University, Xi’an, Shaanxi, P.R. China; 3 Department of Respiratory Medicine, Shaanxi Provincial Second People’s Hospital, Xi’an, Shaanxi, P.R. China; 4 Department of Pathology, Fourth Military Medical University, Xi’an, Shaanxi, P.R. China

**Keywords:** G2/M arrest, advanced non-small cell lung cancer, prognostic biomarkers, molecular pathology

## Abstract

The aim of the present study was to retrospectively assess the correlation between the expression levels of proteins involved in G2/M arrest signaling pathways in non-small cell lung cancer (NSCLC) tissue, as determined by immunohistochemical (IHC) methods, and the overall survival of patients with advanced stage NSCLC. IHC analysis of advanced NSCLC specimens was used to determine the expression levels of proteins involved in G2/M arrest signaling pathways, including ataxia telangiectasia mutated (ATM) kinase, ataxia telangiectasia and Rad3-related (ATR) kinase, checkpoint kinase (Chk) 1, Chk2, cell division cycle 25C (Cdc25C), total cyclin-dependent kinase 1 (Cdk1) and active Cdk1 signaling pathways, the latter of which refers to dephospho-Cdk1 (Tyr15) and phospho-Cdk1 (Thr161). Patients were enrolled continuously and followed up for ≥2 years. Univariate analysis demonstrated that the protein expression levels of dephospho-Cdk1 (P=0.015) and phospho-Cdk1 (P=0.012) exhibited prognostic significance, while the expression of the other proteins was not significantly associated with patient survival (ATM, P=0.843; ATR, P=0.245; Chk1, P=0.341; Chk2, P=0.559; Cdc25C, P=0.649; total Cdk1, P=0.093). Furthermore, the patients with tumors exhibiting low expression levels of active Cdk1 survived significantly longer than those with tumors exhibiting high active Cdk1 expression levels (P<0.05). In addition, Cox regression analysis demonstrated that the expression of active Cdk1 [odds ratio (OR), 0.624; 95% confidence ratio (CI), 0.400–0.973; P=0.038] and the pathological tumor-node-metastasis stage (OR, 0.515; 95% CI, 0.297–0.894; P=0.018) were significant independent prognostic factors for NSCLC. Therefore, the results of the present study indicated that active Cdk1 protein is an independent prognostic factor for advanced NSCLC and may validate Cdk1 as a therapeutic target for advanced NSCLC patients.

## Introduction

Lung cancer is the most common cause of cancer-related mortality worldwide, accounting for 17% of all cancer mortalities (1). Non-small cell lung cancer (NSCLC) is the predominant type of lung cancer, which mainly includes squamous cell carcinoma, large cell carcinoma and adenocarcinoma (2). Surgery is the first choice of treatment for early-stage NSCLC, while chemotherapy and radiotherapy are often administered to advanced NSCLC patients (3). However, the majority of advanced-stage NSCLC patients face unsatisfactory outcomes. Targeted molecular therapy has attained good effects in the treatment of NSCLC. However, the major challenges are variable responsiveness and the development of drug resistance (4). Therefore, there is an urgent requirement to find new therapeutic targets for the treatment of NSCLC.

When DNA is damaged, the G2 cell cycle checkpoint prevents cells from entering mitosis, allowing DNA repair to occur and halting the proliferation of damaged cells (5). Additionally, the role of the G2 checkpoint in facilitating the maintenance of genomic stability indicates that it is important in understanding the molecular mechanism of lung cancer. Ataxia telangiectasia mutated (ATM) kinase, and ataxia telangiectasia and Rad3-related (ATR) kinase are two serine/threonine kinases that regulate cell cycle checkpoints and DNA repair in response to exposed DNA double-stranded breaks (6,7). ATM and ATR kinase act upstream of checkpoint kinases (Chk) 1 and 2; ATM/ATR phosphorylates Chk1 at Ser317 and Ser345 (8), and Chk2 at Thr68 and other sites in the amino-terminal domain, in response to blocked DNA replication, particularly when caused by DNA double-stranded breaks (9). Activated Chk1/2 then exerts its checkpoint mechanism on the cell cycle, in part, by regulating the cell division cycle 25 (Cdc25) family of phosphatases, inactivating Cdc25C via phosphorylation at Ser216, thus preventing the activation of cyclin-dependent kinase 1 (Cdk1) and the transition of the cell into mitosis (10). The entry of all eukaryotic cells into mitosis is regulated by the activation of Cdk1 at the G2/M transition. Cdk1 activation is a multi-step process that is initiated by the binding of the regulatory subunit, cyclin B1, to Cdk1 to form the mitosis-promoting factor (MPF) (11). MPF remains in an inactive state until the phosphorylation of Cdk1 at Thr161 by Cdk activating kinase (CAK) (12) and the dephosphorylation of Cdk1 at Thr14/Tyr15 by phosphatase Cdc25C (13); thus, active Cdk1 refers to dephospho-Cdk1 (Tyr15) and phospho-Cdk1 (Thr161). Furthermore, active Cdk1 facilitates the smooth transition of lung cancer cells from the G2 phase to the M phase, and promotes cell growth and proliferation. Therefore, it has been proposed that the ATM/ATR-Chk1/2-Cdc25C-Cdk1/cyclin B1 signaling pathway is important in G2/M arrest in response to DNA damage in lung cancer. The present study was performed to retrospectively assess the effects of the expression levels of G2/M signaling pathway proteins in NSCLC tissues, as determined by immunohistochemical (IHC) methods, on the prediction of the overall survival (OS) of advanced-stage NSCLC patients.

## Patients and methods

### Patients and tissue specimens

Consecutive patients with pathological stage III and IV squamous cell carcinoma (SCC) or adenocarcinoma (ADC) were retrospectively enrolled in the present study from two university hospitals (Xijing Hospital and Shaanxi Provincial Second People’s Hospital) in Xi’an, China. Patients who received docetaxel and cisplatin doublet chemotherapy for >2 cycles and standard supportive care between June 2008 and July 2011 were qualified for involvement in the present study. Tumors were staged according to the seventh edition of the International System for Staging Lung Cancer developed by the American Joint Committee for Cancer (14), and histopathological tumor types were determined according to the classification of the World Health Organization (15). The inclusion criteria were as follows: i) Tumor unsuitable for surgical removal; ii) availability of formalin-fixed, paraffin-embedded primary lung cancer tissue blocks; and iii) availability of OS data. Patients who had previously received <2 cycles of docetaxel and cisplatin doublet chemotherapy, had previously experienced severe liver and kidney dysfunction or had a history of other types of cancer were excluded from the present study. To increase the quality of the data obtained, the reports of the tissue pathology, the radiological examinations, including chest computed tomography scans and positron emission tomography, and the clinical information for all patients were independently reviewed. The scientific study committee of Xijing Hospital (Xi’an, China) for pulmonary diseases reviewed and approved the database. Consent was obtained from the families of the patients.

### IHC staining

IHC analysis was conducted using BOND-MAX™ (cat. no. M 211-64; Leica Microsystems Ltd., Wetzlar, Germany), a fully automatic IHC and *in situ* hybridization machine. From the prepared sections, one section containing the maximum amount tumor tissue and minimal or absent necrosis and hemorrhage was selected for each patient. The selected sections were deparaffinized via a graduated alcohol and xylene series followed by rehydration in distilled water. Antigen retrieval was performed by adding citrate buffer (pH 6.0) and heating in a microwave oven for 20 min at 100°C. The sections were subsequently incubated in a 3% hydrogen peroxide solution to block endogenous peroxidase activity and then washed with a phosphate-buffered saline solution. Following incubation with blocking solution for 20 min, the sections were incubated again with primary and then secondary antibodies at appropriate dilutions. Cdk1 requires Thr14/Tyr15 dephosphorylation by phosphatase Cdc25C and Thr161 phosphorylation by CAK to transition from an inactive to active protein (16), therefore, the present study used a rabbit monoclonal anti-Cdk1 antibody (dephospho Cdk1 Tyr15; cat no. ab32384; dilution, 1:200) to stain the Cdk1 protein without phosphorylation of Tyr15, a rabbit polyclonal anti-Cdk1 antibody (phospho-Thr161; cat no. ab47329; dilution, 1:100) to stain phospho-Cdk1, and a mouse anti-Cdk1 monoclonal antibody (total Cdk1; cat no. ab8040; dilution, 1:200) to stain the total Cdk1 protein (all Abcam, Cambridge, UK). The reaction was visualized using a 3,3′-diaminbenzidine substrate system (cat. no. 08102; Leica Microsystems Ltd.) and counterstaining was performed using Mayer’s hematoxylin. All of the aforementioned procedures were performed in accordance with the antibody manufacturer’s instructions.

### Evaluation of protein expression level

**Figure 1:**
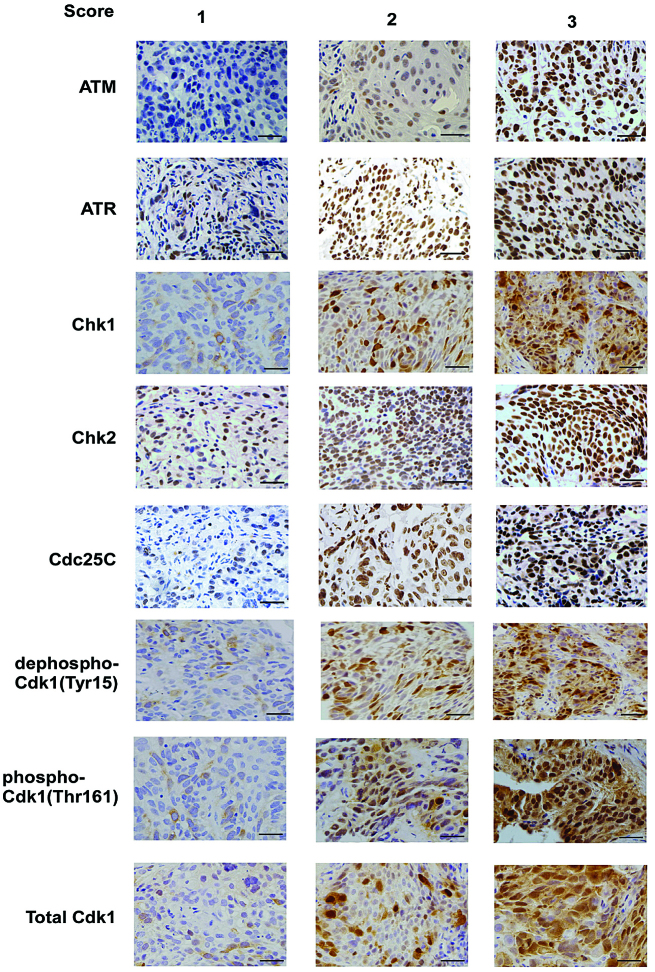
Examples of protein expression scores by immunohistochemistry. Scores correspond to protein expression levels: 1, weak; 2, moderate; 3, strong. Scale bar, 20 μm; magnification, ×400.

Two pathologists who were blinded to the clinical data independently evaluated the IHC staining. The staining intensity of each protein in the cancer cells was graded on a scale of 1–3 (1, weak; 2, moderate; and 3, strong) (Fig. 1), and the percentage of cancer cells positive for each protein was determined and assigned as a proportion score (0, 0%; 0.1, 1–9%; 0.5, 10–49%; and 1.0, ≥50%), as previously described (17). The intensity and proportion scores were then multiplied to yield the semiquantitative H-score. The median value of all the mean H scores was selected as the cutoff value for each protein to separate the cancer cells with high and low expression levels (18). Furthermore, OS was calculated using the day of the lung cancer diagnosis as the first day and the day of mortality as the final day.

### Statistical analysis

The cases were evaluated for demographic and pathological variables, and the expression of the proteins was dichotomized as low versus high. Patient cumulative survival was analyzed using the Kaplan-Meier method, with the date of pathological diagnosis defined as time zero and mortality as the end-point. Differences in survival were determined by performing a log-rank test in the univariate analyses and by using a Cox proportional hazards regression model with backward Wald for prognostic factors in the multivariate analyses. All analyses were performed using SPSS software (version 13.0; SPSS, Inc., Chicago, IL, USA) and P<0.05 was considered to indicate a statistically significant difference.

## Results

### Patient characteristics and univariate analysis

**Figure 2:**
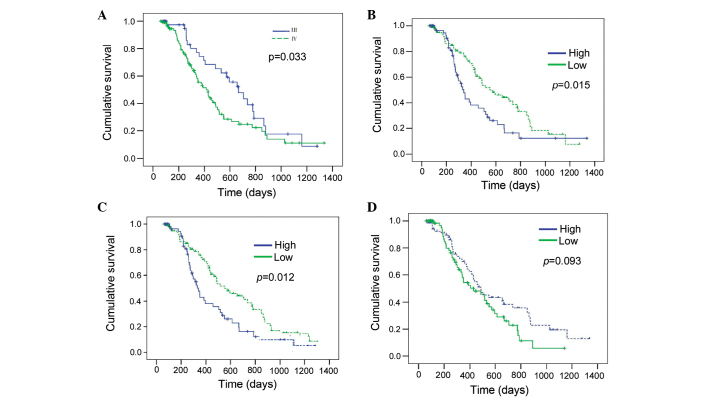
Kaplan-Meier survival curves of advanced non-small cell lung cancer patients with (A) different tumor-node-metastasis stages (III and IV); and different expression levels of (B) dephospho-cyclin-dependent kinase (Cdk) 1 (Tyr 15); (C) phospho-Cdk1 (Thr 161); and (D) total Cdk1 protein.

Table I indicates the clinical characteristics of the 144 patients in the present study, including 64 cases of squamous cell carcinoma, 69 cases of adenocarcinoma and 11 cases of other types of NSCLC. The median age of the patients was 58.4 years. The patients included 109 males and 35 females, 93 (64.58%) of whom were ever-smokers and 51 (35.42%) of whom were never-smokers. The predominant pathological tumor-node-metastasis (TNM) stages of the patients were stage III (45 patients; 31.25%) and stage IV (99 patients; 68.75%). Furthermore, according to univariate analysis, determined by log-rank test, the parameters of age, gender, smoking habit, histology, tumor size (T) and extent of lymphatic metastasis (N) were not significantly associated with the survival of the patients with advanced NSCLC (Table I); however, as expected, the pathological TNM stage was a significant prognostic factor. The one-year survival rate was 74.20% for stage III patients (median survival, 666 days) and 55.60% for stage IV patients (median survival, 415 days) (P=0.033; Table I). Additionally, stage III patients exhibited a more favorable prognosis compared with stage IV patients (Fig. 2A).

### Prognostic value of proteins involved in the G2/M arrest signaling pathway

According to the univariate analysis determined by log-rank test, dephospho-Cdk1 (Tyr15; P=0.015) and phospho-Cdk1 (Thr161; P=0.012) exhibited prognostic significance, while the other proteins exhibited no significant difference in patient survival (ATM, P=0.843; ATR, P=0.245; Chk1, P=0.341; Chk2, P=0.559; Cdc25C, P=0.649; total Cdk1, P=0.093) (Table I; Fig. 2B–D). Kaplan-Meier survival analysis indicated that high expression levels of dephospho-Cdk1 (Tyr15) and phospho-Cdk1 (Thr161) correlated with a worse prognosis in the advanced NSCLC patients, whereas the patients with tumors exhibiting low dephospho-Cdk1 (Tyr15) and phospho-Cdk1 (Thr161) expression levels exhibited a more favorable prognosis (Fig. 2B and C). Furthermore, the one-year survival rates of the patients were 42.90% for high dephospho-Cdk1 (Tyr15) expression (median survival, 339 days) and 74.00% for low dephospho-Cdk1 (Tyr15) expression (median survival, 552 days). The same trend was observed in phospho-Cdk1 (Thr161), with one-year survival rates of 41.32% for high phospho-Cdk1 (Thr161) expression (median survival, 334 days) and 76.78% for low expression (median survival, 562 days) (Table I; Fig. 2B and C). Factors that were determined to affect the survival rate in the univariate analysis (smoking habit, N classification, pathological TNM stage and Cdk1 expression) were analyzed in a multivariate Cox regression analysis of factors that may affect the survival rate. This analysis demonstrated that the expression of dephospho-Cdk1 [Tyr15; odds ratio (OR), 0.619; 95% confidence interval (CI), (0.458–0.925); P=0.032] and phospho-Cdk1 (Thr161; OR, 0.631; 95% CI, 0.412–0.961; P=0.026) were independent prognostic factors of NSCLC (Table II). In addition, the prognostic role of Cdk1 in advanced NSCLC was validated by combining the expression levels of dephospho- and phospho-Cdk1; Cox regression analysis of this variant (active Cdk1) determined that active Cdk1 expression (OR, 0.624; 95% CI, 0.400–0.973; P=0.038) was also an independent prognostic factor of NSCLC (Table II). As expected, the pathological TNM stage (OR, 0.515; 95% CI, 0.297–0.894; P=0.018) was identified as an independent prognostic factor, however, smoking habit and N classification exhibited no significance with regard to the prognosis of NSCLC (Table II).

## Discussion

Early-stage NSCLC patients typically exhibit a high five-year survival rate following curative surgery plus adjuvant chemotherapy and radiotherapy (19). However, numerous patients with advanced NSCLC (stages III and IV) succumb quickly due to disease relapse, despite the administration of a combination of multidisciplinary treatments (20). Pathological TNM staging aids in the prediction of the OS of a group of patients, however, it cannot provide a molecular target for subsequent treatment. Thus, independent prognostic molecular markers, which may additionally serve as treatment targets, must be identified for advanced NSCLC. A number of studies for molecular-targeted treatment in advanced NSCLC have been successful. For example, epidermal growth factor (EGFR) mutations (21) and anaplastic lymphoma kinase (ALK) rearrangements (22) were identified as independent prognostic factors for advanced NSCLC, thus, EGFR tyrosine kinase inhibitors (23) and ALK inhibitors (24) were developed to target these two genes, and proved successful in the treatment of advanced NSCLC. The present study included patients with advanced-stage tumors who were treated with multidisciplinary modalities. Additionally, factors that may affect the prognosis of a group of patients, such as age, gender, smoking habit, N classification, pathological TNM stage and histological type, were included in the statistical analysis. This was expected to reveal the value of G2/M signaling pathway proteins as prognostic biomarkers of advanced NSCLC.

In the present study, univariate analysis determined that age, gender, smoking habit, histology, and T and N classification were not significantly associated with survival in the advanced NSCLC patients. This may be attributed to the advanced stage (stage III and IV) of the patients included in the present study, which diminishes the effect of these factors on patient prognosis. However, pathological TNM stage remained a strong prognostic factor (P=0.033; Table I) and Cox regression analysis demonstrated that pathological TNM stage was an independent prognostic factor for the advanced NSCLC patients (P=0.018; Table II); this prognostic significance may indicate the reliability of the current study.

In addition, univariate analysis demonstrated that the protein expression levels of ATM, ATR, Chk1, Chk2, Cdc25C and total Cdk1 were not significantly associated with a difference in the survival of advanced NSCLC patients (P>0.05). The prognostic role of a number of the aforementioned proteins has previously been studied in early-stage NSCLC. For example, Choi *et al* (25) reported that the protein expression level of ATM and Chk2 had no effect on the OS of stage I NSCLC patients, and Grabauskiene *et al* (26) identified that elevated Chk1 expression in early-stage primary lung adenocarcinoma (442 resected specimens) correlated with poor tumor differentiation and significantly diminished patient survival. However, the prognostic role of the Chk1 expression level could not be validated in the present study, possibly due to the advanced stage of the included NSCLC patients. Wu *et al* (27) analyzed primary tumors and corresponding healthy lung tissues from 40 NSCLC patients and reported no Cdc25C overexpression and no association with patient survival. Furthermore, Abdulkader *et al* (28) investigated a series of 205 carcinomas of the large bowel, breast, lung and prostate, and determined that Cdk1 expression was not associated with the prognosis of early-stage NSCLC. Cdk1 is located at the end of the G2/M signaling pathway, and is therefore key in the G2/M arrest and cell apoptosis induced by chemotherapy and radiotherapy in tumor cells (29). Therefore, to identify the prognostic role of Cdk1 protein, active dephospho-Cdk1 (Tyr15) and phospho-Cdk1 (Thr161) were investigated.

In the present study, the log-rank test identified that dephospho-Cdk1 (Tyr15) and phospho-Cdk1 (Thr161) exhibit prognostic significance in advanced NSCLC patients. In addition, the Cox regression model revealed active Cdk1 to be an independent prognostic factor for NSCLC patients. Patients with high active Cdk1-expression tumors exhibited a significantly shorter survival time compared with low active Cdk1-expressing tumors, indicating that advanced NSCLC patients may benefit from Cdk1 inhibitory treatment. In the cell cycle, Cdk1 is a master modulator of initiation and transition through mitosis, with high active Cdk1 expression levels able to promote G2/M transition and accelerate tumor cell growth (30). Previous studies have demonstrated that decreased phospho-Cdk1 (Thr161) expression (30), as well as the accumulation of phospho-Cdk1 (Tyr15) (31), are involved in G2/M arrest and apoptosis in lung cancer, thus validating Cdk1 as a possible therapeutic target. Furthermore, Vassilev *et al* (32) previously identified a selective small-molecule inhibitor of Cdk1 that reversibly arrests human cells at the G2/M phase and induces apoptosis in tumor cells, indicating that selective Cdk1 inhibitors may have potential clinical utility in cancer therapy. Moreover, Cdk1-regulated G2/M arrest and cell apoptosis are involved in the molecular mechanisms of a number of chemotherapeutic agents and radiotherapy regimens (33); thus, Cdk1 inhibitors may serve to sensitize cells to chemotherapy and radiotherapy in cases of advanced NSCLC that are specifically resistant to conventional treatment strategies.

In conclusion, the OS of a patient with advanced NSCLC appears to depend on numerous factors. The present study indicates that active Cdk1 protein is an independent prognostic factor for advanced NSCLC, with high active Cdk1-expressing tumors correlating with a poor prognosis compared with low active Cdk1-expressing tumors. These results may validate the use of Cdk1 as a therapeutic target for advanced NSCLC patients.

## Figures and Tables

**Table I:** Univariate analysis of prognostic factors in advanced non-small cell lung cancer patients (n=144).

Parameter	Patients, n (%)	One-year survival rate, %	Median survival, days	P-value^a^
Gender				0.676
Male	109 (75.69)	62.30	465	
Female	35 (24.31)	58.30	430	
Smoking habit				0.608
Never	51 (35.42)	62.40	490	
Ever	93 (64.58)	62.40	465	
Histology				0.527
Squamous cell	64 (44.44)	59.90	430	
Adenocarcinoma	69 (47.92)	61.90	552	
Other	11 (7.64)	55.60	389	
T classification				0.998
T1	12 (8.34)	50	350	
T2	48 (33.33)	62.30	487	
T3	25 (17.36)	72.60	503	
T4	59 (40.97)	58.80	437	
N classification				0.173
N0	38 (26.38)	66.50	550	
N1	13 (9.03)	66.70	773	
N2	62 (43.06)	56.20	428	
N3	31 (21.53)	60.40	430	
Pathological TNM stage				0.033
III	45 (31.25)	74.20	666	
IV	99 (68.75)	55.60	415	
ATM expression				0.843
High	66 (45.83)	57.00	389	
Low	78 (54.17)	64.20	514	
ATR expression				0.245
High	60 (41.67)	58.90	415	
Low	84 (58.33)	63.00	503	
Chk1 expression				0.341
High	90 (62.50)	71.50	487	
Low	54 (37.50)	58.50	465	
Chk2 expression				0.559
High	100 (69.44)	62.20	465	
Low	44 (30.56)	59.90	457	
Cdc25C expression				0.649
High	97 (67.36)	61.50	465	
Low	47 (32.64)	60.60	457	
Total Cdk1 expression				0.093
High	71 (49.31)	68.10	487	
Low	73 (50.69)	54.20	430	
Dephospho-Cdk1 (Tyr15) expression				0.015
High	64 (44.44)	42.90	339	
Low	80 (55.56)	74.00	552	
Phospho-Cdk1 (Thr161) expression				0.012
High	65 (45.14)	41.32	334	
Low	79 (54.86)	76.78	562	

a Determined by univariate Kaplan-Meier analysis. Mean age of patients, 58.5 years (range, 31–74 years).

TNM, tumor-node-metastasis; ATM, ataxia telangiectasia mutated kinase; ATR, ataxia telangiectasia and Rad3-related kinase; Chk, checkpoint kinase; Cdc, cell division cycle; Cdk, cyclin-dependent kinase.

**Table II:** Prognostic significance of pathological TNM stage and Cdk1 expression revealed by multivariate analyses in advanced non-small cell lung cancer patients (n=144).

	Multivariate P-value	Odds ratio	95% confidence incidence
Smoking habit	0.066	0.619	0.372–1.033
N classification^a^	0.094		
N1	0.441	0.648	0.215–1.950
N2	0.104	1.569	0.912–2.700
N3	0.07	1.909	0.949–3.838
Pathological TNM stage	0.018	0.515	0.297–0.894
Dephospho-Cdk1 (Tyr15) expression	0.032	0.619	0.458–0.925
Phospho-Cdk1 (Thr161) expression	0.026	0.631	0.412–0.961
Active Cdk1 expression	0.038	0.624	0.400–0.973

a Referenced with N0.

TNM, tumor-node-metastasis; Cdk, cyclin-dependent kinase.
